# Large Symptomatic Splenic Pseudocyst: Case Report of a Rare Entity

**DOI:** 10.1002/ccr3.72584

**Published:** 2026-05-01

**Authors:** Tasnuva Habib Neha, Mobin Ibne Mokbul, Farjana Yesmin, Amrita Shrestha

**Affiliations:** ^1^ Sylhet MAG Osmani Medical College Hospital Sylhet Bangladesh; ^2^ Dhaka Medical College Hospital Dhaka Bangladesh; ^3^ Division of Gastroenterology & Hepatology Mayo Clinic Rochester Minnesota USA; ^4^ Silverline Hospital, Lekhnath Marg Kathmandu Nepal

**Keywords:** abdominal pain, laparoscopic decompression, non‐parasitic splenic cysts, splenic pseudocyst

## Abstract

Large symptomatic splenic pseudocysts, though rare, should be considered in patients with persistent left upper abdominal pain as we report in our case. Imaging enables diagnosis, and laparoscopic spleen‐preserving cyst decompression and deroofing provide a safe, effective treatment, relieving symptoms while maintaining splenic function and avoiding the morbidity associated with splenectomy and its long‐term complications.

## 1 Introduction

Splenic cyst is a very rare clinical entity with an incidence of 0.07% in a review of over 42,000 autopsies [1]. They are often asymptomatic with incidental discovery, but once identified, several diagnostic possibilities may be narrowed down based on the clinical history, imaging appearances, and serological tests [2]. The majority of such splenic cysts have parasitic origin with *Echinococcus granulosus* resulting in a hydatid cyst, whereas only less than one third of cases belong to the non‐parasitic category [3, 4]. Classification of splenic cysts is heterogeneous, mainly classified as primary/true (those with epithelial lining) and secondary/false/pseudo cyst (without epithelial lining) [5]. About 75% pseudo cysts are due to trauma, and the rest are due to infection (tuberculosis, infectious mononucleosis, malaria), degenerative disease, or infarction [6, 7]. Presentation of splenic cyst is variable, ranging from being asymptomatic to development of pressure effect on adjacent viscera, including compression of renal arteries leading to systemic hypertension; rupture to other organs; spontaneous cutaneous fistulization, or segmental portal hypertension [8]. Radiological workup, ranging from ultrasonography (US), computerized tomography (CT) scan, magnetic resonance imaging (MRI), Doppler US, percutaneous US‐assisted cyst puncture, and fluid analysis up to histopathological evaluation, has been utilized in differentiating the type of splenic cyst and formulating effective management strategies [9].

The greatest revolution has occurred in the management technique of splenic cyst from classic total splenectomy to current parenchyma‐preserving surgical procedures due to increased understanding of the spleen's role in immunologic function [10, 11]. Management depends upon size, presentation, mass effect, and other factors ranging from cyst aspiration with or without a sclerosing agent, cyst fenestration and drainage, marsupialization, partial splenectomy, up to total splenectomy as needed [12]. Conservative management is usually indicated for asymptomatic cysts with a diameter of less than 5 cm, whereas active surgical management is preferred for symptomatic cysts or if the cyst is larger than 5 cm [13]. Recurrence following management of cyst by either conservative or surgical approach is more common in true cysts adjacent to other structures (e.g., diaphragm) because of possible epithelization and fluid re‐accumulation or an internally located cyst because of possible incomplete cyst removal [14].

Here, we present a rare case of an inflammatory splenic pseudocyst without traumatic evidence. Excluding the infectious origin of the cyst via ELISA (Enzyme‐linked Immunosorbent Assay), diagnosis was approached through laparoscopy. Aspiration was done on the same setting, followed by diathermic dissection and deroofing of the cyst. Follow‐up after 6 months revealed a normal‐sized spleen without evidence of cyst recurrence. Thus, early identification and management of the pseudocyst can decrease morbidity and mortality resulting from complicated cysts, helping to preserve the spleen.

## 2 Case History/Examination

A 22‐year‐old female of Bengali ethnicity presented with chronic left subcostal pain for 9 months. She had been experiencing intermittent dull aching pain in the left upper abdomen that increased with movement. She also complained of early satiety. On examination, her left costal margin was slightly elevated, but there was no palpable mass in the abdomen. Her vitals were normal, and she had previously been in good health.

## 3 Differential Diagnosis, Investigations and Treatment

**FIGURE 1:**
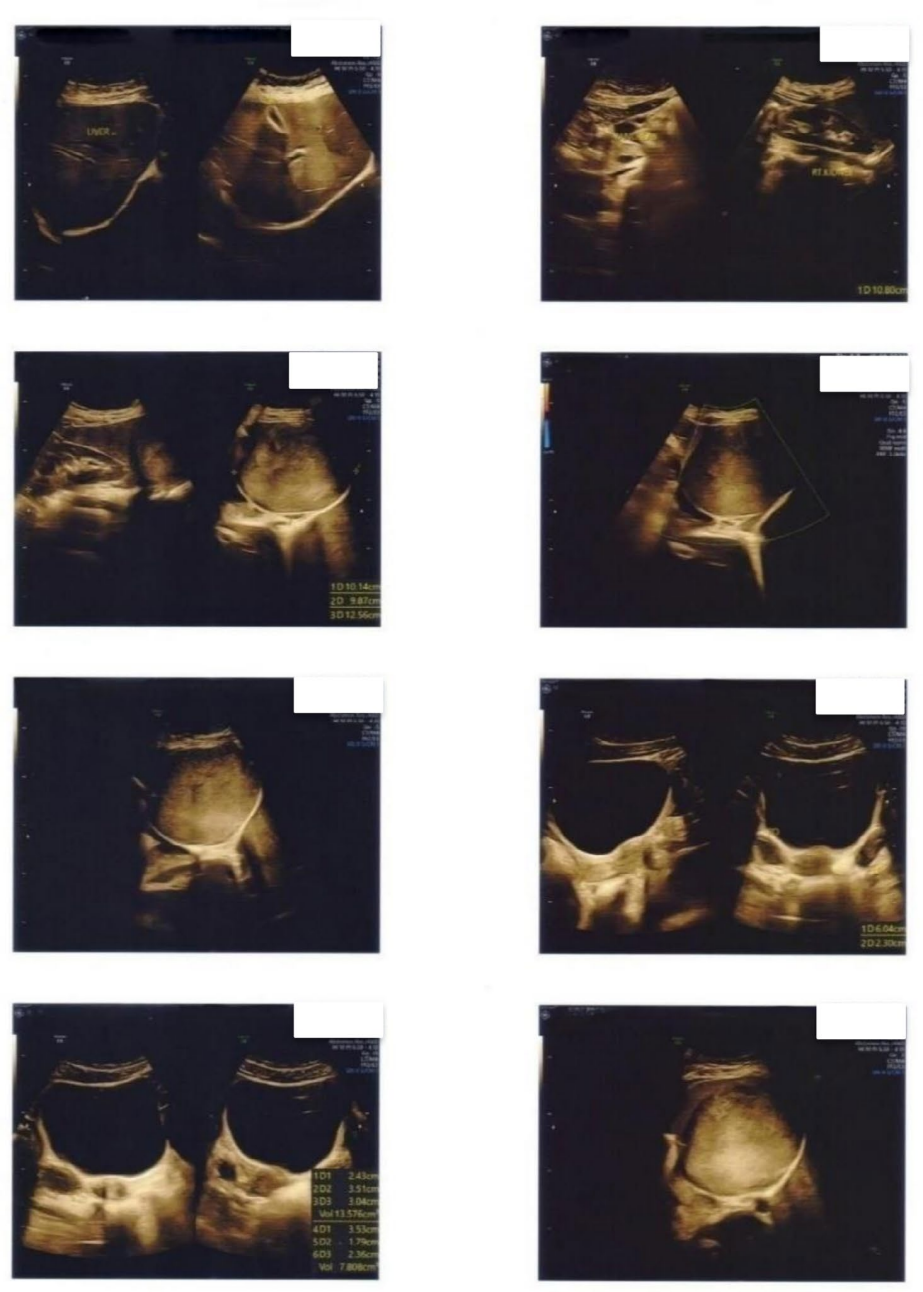
USG of the abdomen showing a large soft tissue echogenic lesion in the left lumbar region.

On abdominal USG, a large soft echogenic lesion (measuring about 12.5 × 9.8 cm) was noted in the left lumbar region related to splenic parenchyma (Figure 1). The lesion was separated from the left kidney.

**TABLE 1:** Overview of splenic pseudocysts.

Etiology	Usually post‐traumatic origin or secondary to hematoma, infarction, infection, orinflammation [14]
Incidence	Only 800–850 cases have been reported till now in the literature
Gender ratio	No gender predominance was found in the case of splenic pseudocyst, but a slight female predominance in the prevalence of splenic cyst according to some case series [1, 9, 15, 16, 17, 18, 19]
Association	Pancreatic cyst, Congenital anomalies like splenoptosis, Sickle cell disease, Infectious diseases like Tuberculosis, Malaria, Infectious mononucleosis, *Treponema pallidum* infection, etc. [1, 9, 15, 16, 17, 18, 19]
Investigation	Imaging like USG, CT scan, MRI, MRA
Management	Conservative & follow up in case of less than 5 cm and symptomatic cysts Surgical intervention is indicated in cases of cysts exceeding 5 cm and symptomatic cysts [1, 9, 15, 16, 17, 18, 19]

Abbreviations: CT‐scan, computed tomography scan; MRA, magnetic resonance angiogram; MRI, magnetic resonance imaging; USG, ultrasonography.

**FIGURE 2:**
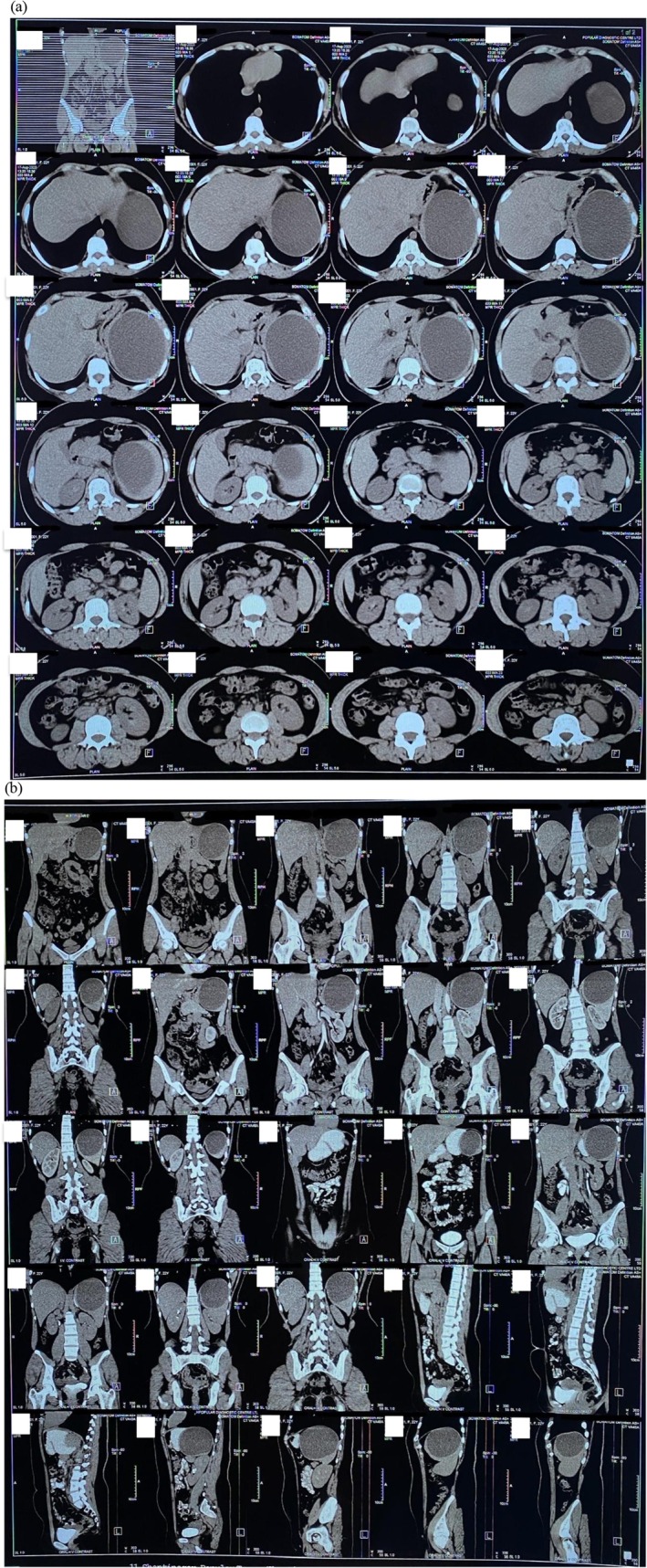
Plain and contrast (a) transverse section and (b) coronal section CT scan of abdomen showing a large cyst in the upper pole of spleen measuring 9.6 cm in AP, 12.2 cm in TR & 11.8 cm in CC. Note that there is no post‐contrast enhancement.

Her abdominal Computed Tomography (CT) scan revealed a large cystic lesion in the spleen having hyper‐attenuated materials within measuring about 9.6 cm in AP, 12.2 cm in TR & 11.8 cm in CC at the upper pole of the spleen (Figure 2). There was no enhancement in the post‐contrast scan.

## 4 Conclusion and Results (Outcome and Follow‐Up)

**FIGURE 3:**
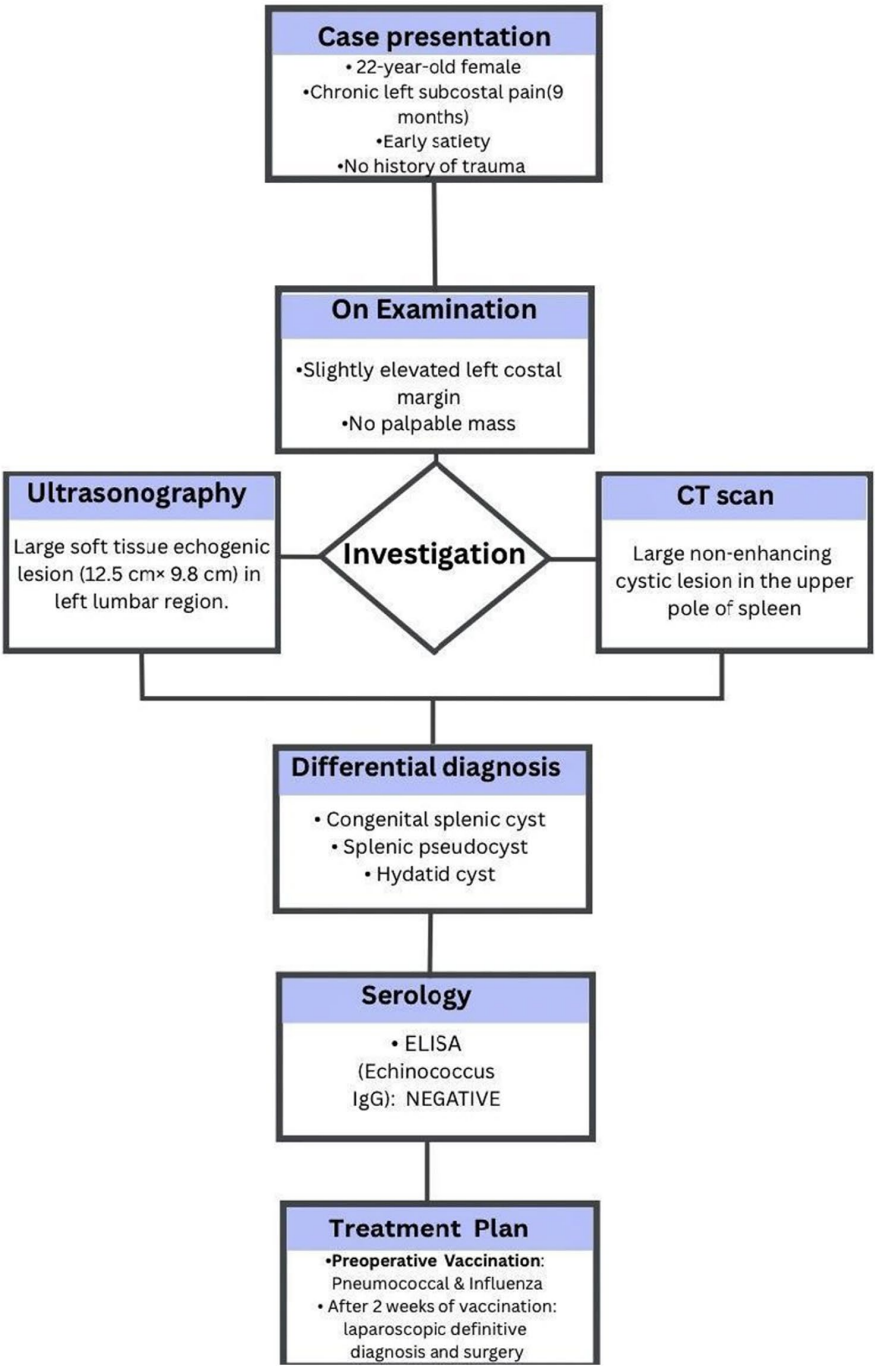
A flowchart showing diagnosis, investigation and management plan in this case of splenic pseudocyst. The patient's party was consulted about all possible treatment options. Depending upon the intraoperative conditions, consent for both laparotomy and splenectomy was also obtained. Two weeks before the surgery, pneumococcal and influenza vaccinations were done. A laparoscopic spleen‐preserving surgery was performed (Figure 3). Decompression was done by aspiration of the serous collection from the cyst. About 600 mL of fluid was collected. Then, a small portion of the cyst was excised by diathermic dissection, and de‐roofing of the cyst was done. Then the momentum was kept in the cystic cavity (known as ‘omental plugging’). Hemostasis was secured with minimal blood loss, and a normal saline wash was given. Histopathological study revealed it was an inflammatory pseudocyst, as the wall was devoid of epithelium. There was some granulation tissue, RBC, and a few hemosiderin‐laden macrophages. No granuloma or malignancy was seen (Figure 4).

The possible diagnoses of such a cystic splenic mass were congenital splenic cyst, hydatid cyst, and splenic pseudocyst. Echinococcus IgG was tested by ELISA, which came out negative. Based on the radiological findings and serological test, the possibility of it being a hydatid cyst was excluded. So the lesion was suspected to be a congenital cyst or pseudocyst, which could be confirmed by histopathology. Figure 3 outlines the complete clinical procedures.

**FIGURE 4:**
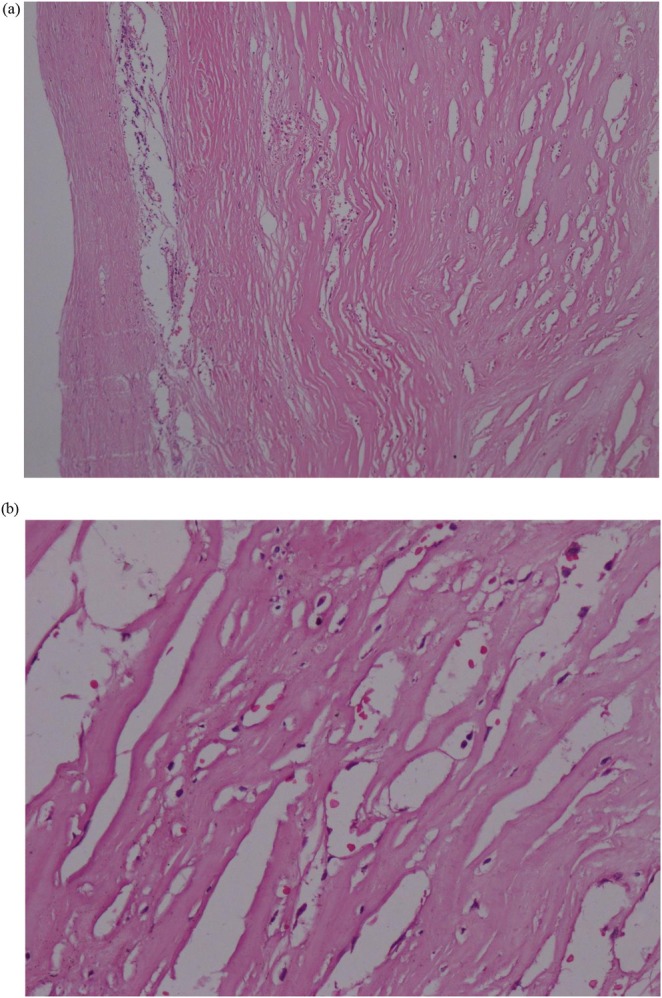
Histopathological study of the cyst wall at (a) 10X and (b) 100X. It shows the cyst is devoid of an epithelial lining, which confirms the diagnosis of splenic pseudocyst. Some granulation tissue, hemosiderin‐laden macrophages, and a small number of RBC can be seen.

The postoperative course was uneventful, and she was released from the hospital on the second postoperative day. She had a complete recovery, and follow‐up ultrasonography after 6 months revealed no abnormalities or recurrence, and the spleen was found to be normal in size.

## 5 Discussion

Splenic pseudocysts are a rare occurrence, found in < 1% of splenectomies (Table 1). To date, only 800–850 cases have been reported in the medical literature [14]. According to the epithelial lining, splenic cysts can be categorized into true or false cysts. True cysts can be further subdivided into parasitic and non‐parasitic cysts, whereas false cysts can be classified into traumatic and non‐traumatic cysts [20]. A recent classification of non‐parasitic splenic cysts includes congenital, traumatic, neoplastic, & degenerative types according to their pathophysiology [21].

Splenic pseudocyst is often asymptomatic. Sometimes, patients experience abdominal pain, distension, and swelling. This presentation can vary depending on other associated conditions like pancreatic pseudocyst, sickle cell disease, infectious diseases, etc. [15, 16]. In our case, the patient complained of chronic left subcostal pain with no history of trauma and no sign of infection.

The Investigation lineup to confirm a splenic cyst includes imaging modalities like USG, CT scan, MRI, and MRA. These techniques help to differentiate between cystic lesions. In our case, initially, USG and later a CT scan confirmed a cystic lesion in the spleen. Given the CT findings, some differential diagnoses like splenic cyst, pseudocyst, hydatid cyst, abscess, and hematoma were considered. A hydatid cyst was excluded through negative results on an ELISA for *Echinococcus* IgG. A histopathological study is necessary, and the gold standard to differentiate between true and pseudocysts, which was also done in our case. In case of association with other complications, exploratory laparotomy may be required for further evaluation and an appropriate management strategy.

Management modalities of splenic pseudocyst depend on its size, location, and associated conditions. In case of cysts less than 5 cm in asymptomatic patients, conservative treatment and regular follow‐up are typically recommended. However, in cysts more than 5 cm in patients exhibiting symptoms, it is more justified to opt for surgical interventions [17].

Various surgical techniques, including marsupialization, deroofing, fenestration of the cyst, spleen‐preserving surgery, or total splenectomy, are often performed. Associated omental repair by omental plugging and omentoplasty may often be necessary.

Splenic pseudocysts have been successfully managed by percutaneous drainage followed by instillation of a sclerosing agent [22]. However, rapid reaccumulation of the cyst has been reported in a case by Lappin J (1992) [23].

Laparoscopic marsupialization is another good surgical technique with less postoperative pain and rapid recovery [24]. It has been reported to be safe even for giant non‐parasitic splenic cysts with more than 15 cm in diameter, and appeared as a safe surgical technique in a case report by Sierra R (2004) [25]. But a high risk of recurrence is still a concern in this approach [26].

Partial cystectomy (fenestration, deroofing) allows resection of only part of the cystic wall, creating a communication between the peritoneal cavity and cystic cavity for drainage. Tagaya N (2007), in a case report, has stated this technique is safe and feasible, as it cured the condition, preserving the splenic tissue with no further complications [27]. But skillful surgical expertise is an absolute prerequisite in this case, as the extent of resection is determined intraoperatively, and there is a high risk of recurrence [28].

Sclerotherapy was stated as a safe and effective method of management in a chronic asymptomatic patient with a splenic pseudocyst. Percutaneous drainage of the cyst followed by administration of ethanol as a sclerosing agent was stated [29]. The safety and efficacy of this method have been mentioned as high in a systematic review and meta‐analysis by Gasparetto A (2023); however, there is a risk of recurrence in cases of cysts more than 8 cm [30].

Due to the immense immunological & hematopoietic functions of the spleen, spleen‐preserving surgery or partial splenectomy has been acknowledged in modern surgical techniques. Specifically, in the case of benign, cystic, or solid lesions, this organ‐preserving surgical technique is more appropriate and feasible. However, the location of the cyst where partial resection is possible, preserving most of the splenic parenchyma, is an important indication in this regard [31]. The most important advantage of this procedure is that the conservation of more than 25% of splenic parenchyma allows regular immunological function [32].

Total splenectomy is required in case of giant, complicated cysts. If the cyst is located near the hilum or has an ill‐defined margin with splenic parenchyma, total splenectomy is indicated [28]. It has also been considered a gold standard technique, considering the high risk of hemorrhage and recurrence in spleen‐preserving surgeries in a case report by Jeenah NR (2023) [33].

A complete guideline for the management of splenic pseudocyst is still unclear, and it is recommended to go for individualized management based on factors like symptoms, size, location & possible postoperative complications. Laparoscopic spleen‐preserving surgery is considered the first goal of management among all the surgical techniques in nonparasitic splenic cysts according to recent literature [28]. However, each case should be evaluated individually, taking into account the specific characteristics of the cysts, imaging results, and the overall patient's health to identify the best surgical intervention.

In our case, laparoscopic spleen‐preserving surgery was performed based on the patient's presentation and imaging results. The procedure involved decompression and deroofing of the cyst.

Overwhelming post‐splenectomy infection (OPSI) is a common complication after partial or total splenectomy. In a review of pertinent English literature, it was found that splenectomized patients are predisposed to overwhelming, fulminant bacterial infections that are often resistant to standard treatment, with a case fatality of 40% to 54% [34, 35].

In our case, the patient complained of no signs of such infections during the 6 months of the post‐operative period to date. The decision to opt for a minimal surgical approach like laparoscopic decompression and deroofing appeared effective in preventing this severe complication.

Another post‐operative consequence is recurrence, which is often a reason for contraindication for most of the minimal surgical approaches and a justification for total splenectomy in such cases. In our case, there has been no recurrence to date. In our opinion, proper imaging techniques and skilled surgical methods are essential to minimize the risk of recurrence in such minimal surgical interventions. Also, the laparoscopic approach of deroofing appears to be an effective strategy for preventing recurrence, as the size and shape can be precisely assessed in the laparoscopic approach compared to the open approach.

In conclusion, splenic pseudocysts are uncommon clinical entities that can exhibit symptoms like stomach pain but are usually asymptomatic. Ultrasound and CT scans are important diagnostic tools, and histology is the gold standard for identifying real cysts from pseudocysts. The treatment of cysts differs according to their size and symptoms. Cysts that are smaller and asymptomatic can usually be treated conservatively, but those that are larger or symptomatic need surgery. Decompression and deroofing are two laparoscopic spleen‐preserving procedures that have demonstrated efficacy for managing these cysts with few complications, lowering the likelihood of severe infections following splenectomy. Following laparoscopic surgery, the patient in this case recovered completely, and a follow‐up revealed no recurrence. This emphasizes the value of personalized treatment and the effectiveness of minimally invasive methods in maintaining splenic function and reducing postoperative complications.

## Author Contributions

**Tasnuva Habib Neha:** conceptualization, data curation, visualization, writing – original draft. **Mobin Ibne Mokbul:** conceptualization, project administration, visualization, writing – original draft, writing – review and editing. **Farjana Yesmin:** writing – original draft. **Amrita Shrestha:** writing – original draft.

## Funding

The authors have nothing to report.

## Ethics Statement

The authors have nothing to report.

## Consent

The patient has been fully anonymized, and written informed consent for publication was obtained.

## Conflicts of Interest

The authors declare no conflicts of interest.

## Data Availability

The authors have nothing to report.
